# Neuro-fuzzy classification of asthma and chronic obstructive pulmonary disease

**DOI:** 10.1186/1472-6947-15-S3-S1

**Published:** 2015-09-11

**Authors:** Almir Badnjevic, Mario Cifrek, Dragan Koruga, Dinko Osmankovic

**Affiliations:** 1 University of Zagreb, Faculty of Electrical Engineering and Computing, Unska 3, 10000 Zagreb, Croatia; 2 Department of Respiratory Diseases, Army Medical Center, 21000 Novi Sad, Serbia; 3 Faculty of Electrical Engineering Sarajevo, University of Sarajevo, 71000 Sarajevo, Bosnia and Herzegovina

## Abstract

**Background:**

This paper presents a system for classification of asthma and chronic obstructive pulmonary disease (COPD) based on fuzzy rules and the trained neural network.

**Methods:**

Fuzzy rules and neural network parameters are defined according to Global Initiative for Asthma (GINA) and Global Initiative for chronic Obstructive Lung Disease (GOLD) guidelines. For neural network training more than one thousand medical reports obtained from database of the company CareFusion were used. Afterwards the system was validated on 455 patients by physicians from the Clinical Centre University of Sarajevo.

**Results:**

Out of 170 patients with asthma, 99.41% of patients were correctly classified. In addition, 99.19% of the 248 COPD patients were correctly classified. The system was 100% successful on 37 patients with normal lung function. Sensitivity of 99.28% and specificity of 100% in asthma and COPD classification were obtained.

**Conclusion:**

Our neuro-fuzzy system for classification of asthma and COPD uses a combination of spirometry and Impulse Oscillometry System (IOS) test results, which in the very beginning enables more accurate classification.

Additionally, using bronchodilatation and bronhoprovocation tests we get a complete patient's dynamic assessment, as opposed to the solution that provides a static assessment of the patient.

## Background

Asthma is an inflammatory impairment of airways, which as result becomes hyperreactive and generates increased mucus, mucosal swelling and contraction of smooth airway muscles, all of which contribute to airway obstruction. In addition, the prevalence of asthma has been increasing in the recent decades, as well as the costs for asthma care [1-3]. One of the problems related to asthma management is that a number of patients with asthma are either improperly diagnosed or misdiagnosed for having other respiratory diseases such as a common cold, acute bronchitis or chronic obstructive pulmonary disease (COPD) [4,5].

COPD is a respiratory disorder characterized by chronic and recurrent airflow obstruction, which increases resistance and dynamic hyperinflation of the peripheral airways [6,7]. The two main examples of this are obstructive emphysema and chronic bronchitis. Approximately 75% of all COPD patients do not have diagnosis. Most of them have a mild degree COPD, but among them a 4% are with severe degree and 1% with very severe degree of COPD [8]. Approximately 200,000 to 300,000 people in Europe die every year due to COPD, and the number exceeds the quote of lung cancer and breast cancer together [9-11].

Based on presented diagnosis problems there is a need for the system which can help physicians to make more successful diagnosis.

The most commonly used pulmonary function tests that are used to detect asthma and COPD are spirometry and Impulse Oscillometry System (IOS) [12,13]. Neuro-fuzzy systems are used for the detection different types of diseases. In 2004, Barua et al [14] presented a system based on trained neural networks that uses the results of measurements performed by IOS for classification of asthma. In 2009, Winkler et al [15] were able to diagnose asthma and COPD patients using different measurement methods on IOS. Asaithambi et al [16] in 2012 made classification of respiratory abnormalities using adaptive neuro fuzzy inference system based only on spirometric measurements. As can be seen from the papers [14-16] the authors have tried to using their neuro fuzzy systems to perform diagnosis of disease based on the results of lung function measurements using only IOS or spirometry. In 2008, Meraz et al [17] designed software for classification of respiratory disease in children. The same year Hafezi [18] made an integrated software package for model-based neuro-fuzzy classification of small airway dysfunction, based on augmented RIC (aRIC) and extended RIC (eRIC) equivalent lung models and IOS data.

The most successful diagnosis is achieved in combination of IOS and spirometry measuring test results [6-9]. Based on it a static assessment of the patient is obtained, which is achieved only on symptoms and first pulmonary function testing, without any further monitoring of patients. In order to get dynamic assessment of the patient, it is necessary to take the patient's symptoms and allergies in consideration, to perform auscultation of the patient, and to apply bronchial dilation (BDT) and bronchial provocation tests (BPT). After BDT and/or BPT treatment and after the second and/or third measurement of lung function, potential changes of pulmonary parameter values may be presented, from which physicians get accurate information on the specifics of the disease. Dynamic assessment of the patient is necessary process according to Global Initiative for Asthma (GINA) and Global Initiative for chronic Obstructive Lung Disease (GOLD) guidelines [19,20].

This paper presents a neuro-fuzzy system for classification of asthma and COPD based on GINA and GOLD guidelines.

## Methods

### Neuro-fuzzy system design

**Figure 1:**
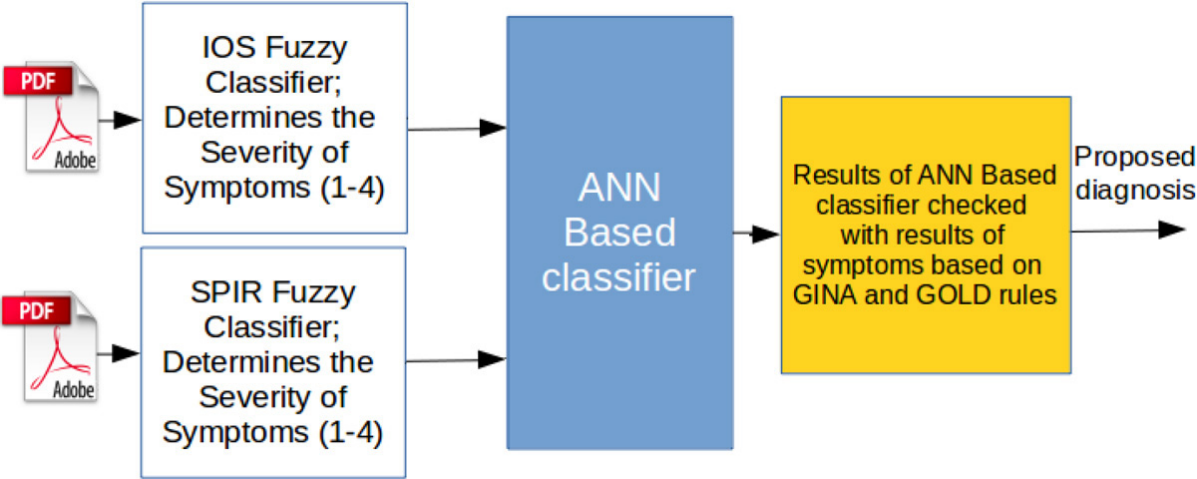
**Architecture for asthma and COPD classification**.

Neuro-fuzzy system architecture for asthma and COPD classification is presented in Figure 1.

Input data for our fuzzy rules are results from .pdf files obtained from spirometry (SPIR) and IOS measurement reports. Those data are used to preliminary perform classification of diseases, and they present input vector of the neural network. Symptoms based on GINA and GOLD guidelines and ANN classification results gives proposed diagnosis.

Asthma was defined based on history of recurrent wheezing, cough, chest tightness or shortness of breath, in accordance to allergic symptoms, which could have a seasonal pattern or might exacerbate at night or by common exposure to allergen, exercise or smoking. Patients with positive symptoms and Resistance at 5 Hz (R5)>150%pred, Resistance at 20 Hz (R20)>150%pred, Reactance at 5 Hz (X5pred - X5)>0.15 kPa/(L/s), Ratio of Forced Expiratory Volume in 1 second and Forced Vital Capacity (FEV1/FVC)<0.8 were diagnosed with asthma. Patients with nonspecific test results were dedicated for bronchial hyper reactivity (BHR), first with BDT, and then with BPT. Patients are diagnosed with asthma when their R5 decreases by more than 25% (ΔR5), the absolute value of X5 decreases by more than 20% (ΔX5) and FEV1 decreases by more than 12% of baseline value and 200 ml. In cases where BDT did not achieve any improvement for final diagnose, patients were examined with methacholine BPT. The doses of methacholine, serially increased, were administered, and FEV1 was measured after each methacholine dose until a 20% decrease in FEV1. After this procedure, the system was able to make the final classification of asthma.

COPD was diagnosed based on positive history of dyspnea (progressive, exertion or persistent), chronic cough (intermittent or non-productive), chronic sputum and a history of exposure to tobacco, occupational dusts, chemicals or other smokes at an age above 40. The diagnosis of COPD was confirmed by having a positive history, R5>150%pred, (X5pred - X5)>0.15 and FEV1/FVC<0.7. Patients with nonspecific test results were evaluated for bronchial hyperreactivity (BHR) by means of BDT, in order to check which group of COPD they belonged to (A, B, C or D) [19]. The bronchial dilation test was performed according to international guidelines using 400 μg of salbutamol. After the completion of the test, the system was able to make the final classification of COPD.

### Fuzzy logic design

Developed fuzzy systems have been designed according to experience of other researchers [21-24] and instructions of GINA and GOLD guidelines.

Input variables for implemented fuzzy system for the case of IOS are values of R5, R20 and X5 which are obtained from IOS test report, after pulmonary function testing's. Their fuzzy values are defined by the equations from 1 to 7.

(1) $$\mu_{normal}\left(R5\right)=\left\{\begin{array}{c} 1,\;\;\;\;\;0<R5<150\\ \frac{R5-145}{5},\;150\leq R5\leq 155\\ 0,\;\;\;\;\;\;R5>155 \end{array}\right)$$

(2) $$\mu_{high}\left(R5\right)=\left\{\begin{array}{c} 0,\;\;\;\;\;0<R5<150\\ \frac{R5-150}{5},\;150\leq R5\leq 155\\ 1,\;\;\;\;\;\;\;R5>155 \end{array}\right)$$

(3) $$\mu_{normal}\left(X5\right)=\left\{\begin{array}{c} 1,\;\;\;\;\;\;\;\;\;X5<0,15\\ \frac{0,155-X5}{0,005},\;0,15\leq X5\leq 0,155\\ 0,\;\;\;\;\;\;\;\;X5>0,155 \end{array}\right)$$

(4) $$\mu_{high}\left(X5\right)=\left\{\begin{array}{c} 0,\;\;\;\;\;\;\;\;X5<0,15\\ \frac{X5-0,15}{0,005},\;0,15\leq X5\leq 0,155\\ 1,\;\;\;\;\;\;\;X5>0,155 \end{array}\right)$$

(5) $$\mu_{none}\left(\text{Δ}R\right)=\left\{\begin{array}{c} 1,\;\;\;\;\;\;\text{Δ}R<0\\ 1-\frac{\text{Δ}R}{0,05},\;0\leq \text{Δ}R\leq 0,05\\ 0,\;\;\;\;\;\text{Δ}R>0,05 \end{array}\right)$$

(6) $$\mu_{min}\left(\text{Δ}R\right)=\left\{\begin{array}{c} 0,\;\;\;\;\;\;\;\;\text{Δ}R<0,05\\ \frac{\text{Δ}R-0,05}{0,075},\;\;0,05\leq \text{Δ}R\leq 0,125\\ \frac{0,125-\text{Δ}R}{0,075},\;\;0,125\leq \text{Δ}R\leq 0,2\\ 1,\;\;\;\;\;\;\;\;\;\text{Δ}R>0,2 \end{array}\right)$$

(7) $$\mu_{sig}\left(\text{Δ}R\right)=\left\{\begin{array}{c} 0,\;\;\;\;\;\;\;\text{Δ}R<0,2\\ \frac{\text{Δ}R-0,2}{0,15},\;0,2\leq \text{Δ}R\leq 0,35\\ 1,\;\;\;\;\;\;\;\text{Δ}R>0,35 \end{array}\right)$$

where: μ_normal_(R5) is membership function of reference values of R5 from IOS, μ_high_(R5) is membership function of upper limit of normal (ULN) values of R5 from IOS, μ_normal_(X5) is membership function of reference values of X5 from IOS, μ_high_(X5) is membership function of upper limit of normal values of X5 from IOS, μ_none_(ΔR) membership function for none difference between R5 and R20, μ_min_(ΔR) is membership function for minimum difference between R5 and R20 and μ_sig_(ΔR) is membership function for significant difference between R5 and R20. Fuzzy values for R20 are the same as for R5. Input values can be calculated by using fuzzy T-norm operator [25].

Based on the following rules, we can define outputs for the diagnosis that can be classified by using IOS report:

1. if (R5 is normal) and (R20 is normal) and (R5-R20 is none) and (X5 is normal) then (output1 is NSCO-SV);

2. if (R5 is normal) and (R20 is normal) and (R5-R20 is minimum) and (X5 is normal) then (output1 is NSCO-DV);

3. if (R5 is normal) and (R20 is normal) and (R5-R20 is significant) and (X5 is normal) then (output1 is NSPO);

4. if (R5 is ULN) and (R20 is ULN) and (R5-R20 is none) and (X5 is normal) then (output1 is CO-SV);

5. if (R5 is ULN) and (R20 is ULN) and (R5-R20 is minimum) or (X5 is normal) or (X5 is ULN) then (output1 is CO-DV);

6. if (R5 is ULN) and (R20 is ULN) and (R5-R20 is significant) and (X5 is ULN) then (output1 is PO);

where NSCO-SV is Negative Sub Central Obstruction - Same Values, NSCO-DV is Negative Sub Central Obstruction - Different Values, NSPO is Negative Sub Peripheral Obstruction, CO-SV is Central Obstruction - Same Values, CO-DV is Central Obstruction - Different Values, PO is Peripheral Obstruction, while output1 is suggestion of classification based on IOS test report.

### Artificial neural network design

**Figure 2:**
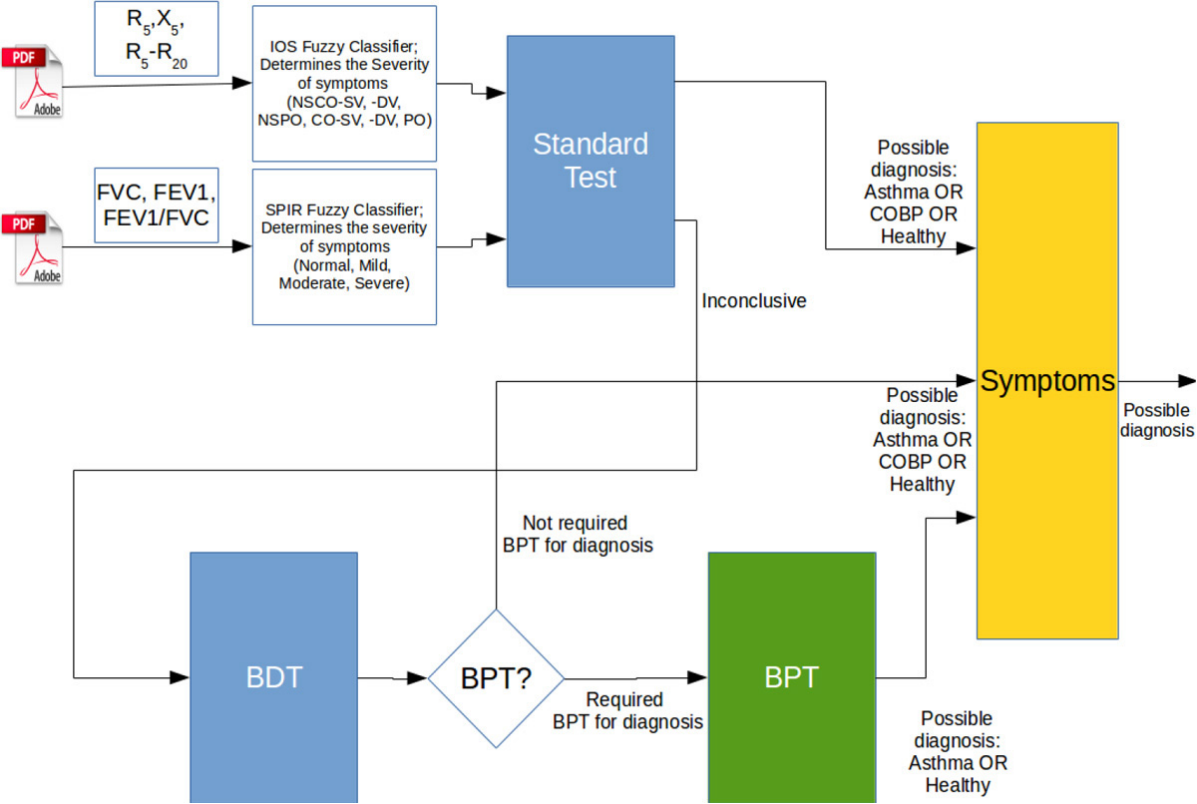
**Data flow of the presented architecture**.

The results of the analysis and classification based on the reports of IOS and spirometry are the input vector of the neural network (NN). Detailed data flow for this architecture is presented in Figure 2.

The linear feed forward (FF) neural network is, according to the application experts, sufficient to properly perform the classification and these types of NN are mostly used for classification task [26-30]. The network is divided into layers. The input layer consists only of network inputs. It is then followed by a hidden layer which consists of a number of neurons, or hidden units placed in parallel. Each neuron performs a weighted summation of the inputs, which is then passed to some non-linear activation function σ, also called the neuron function.

In our case, we choose *tansig *activation function for hidden layer which is equivalent to the hyperbolic tangent function. This function is used in highly non-linear data classification [31] which is true in our case.

The network output is formed by another weighted summation of the outputs of the neurons in the hidden layer. This summation on the output is called the output layer. Generally, the number of output neurons equals the number of outputs of the approximation problem. We choose linear activation function for the output layer, because that is commonly used in regression problems [32].

Artificial neural network (ANN) is trained using estimation data and validated using validation data. We used 80-20 division to get estimation and validation data from the data set. The training data set contains 1000 tests previously obtained from database of the company CareFusion, 800 of them were used for estimation, and other 200 for validation. The training algorithm we used is Levenberg-Marquardt (LMA), which is common training algorithm in data classification [33].

### Neuro-fuzzy system validation

#### Study patients

We included 455 patients in charge of the Clinic for lung diseases at Clinical Centre University of Sarajevo, Bosnia and Herzegovina to test our system of classification of asthma and COPD. Subjects were separated into two groups, healthy and diseased. Diseased subjects were separated into two subgroups, asthmatics and COPD patients. Out of 455 patients, 170 were asthmatics, 248 were COPD, while 37 patients were healthy subjects. Basic information about patients involved in the study are presented in table 1.

**Table 1:** Basic information about patients involved in this study.

Category	**No**.	Age (X±SD)	Gender		Other diseases		Smoking		Allergies		Medications	
			**M **	**F**	**Yes**	**No**	**Yes**	**No**	**Yes**	**No**	**Yes**	**No**

Asthma	170	19.85 ± 8.185	99 58.2%	71 41.8%	43 25.3%	127 74.7%	58 34.1%	111 65.9%	71 41.8%	99 58.2%	88 51.8%	82 48.2%

COPD	248	52.25 ± 7.636	128 51.6%	120 48.4%	179 72.2%	69 27.8%	177 71.4%	71 28.6%	78 31.4%	170 68.6%	162 65.3%	86 34.7%

Healthy	37	30.03 ± 11.833	17 45.9%	20 54.1%	9 24.3%	28 75.7%	22 59.5%	15 40.5%	13 35.1%	24 64.1%	15 40.5%	22 59.5%

Before we began with our study and research we obtained ethics board approval for human subject from Clinical Centre University of Sarajevo. All the volunteers gave written informed consent.

#### Procedure

For purpose of this study, baseline assessment for all patients was to answer on questions regarding symptoms, allergies, history and risk factors of asthma and COPD according to GINA and GOLD guidelines to physicians.

In another step, lung function was measured incorporating spirometry and IOS test results. All pulmonary function test reports were obtained using the CareFusion Germany "Master Screen IOS" device, which allows performing spirometric and impulse oscillometric lung function tests.

Using spirometry, forced vital capacity (FVC) and forced expiratory volume in one second FEV1 were derived, while the ratio FEV1/FVC was calculated. The impulse oscillometry system (IOS) provided respiratory resistance R5 at 5 Hz, proximal resistance R20 at 20 Hz, lung reactance X5 at 5 Hz and resonant frequency Fres [34,35]. All measured results were compared to predicted (pred) values of the patient.

If the patients are with nonspecific test results then it is necessary to do BDT and/or BPT. The bronchial dilation test was performed according to international guidelines [36,37] using 400 μg of salbutamol. In cases where BDT did not achieve any improvement for final diagnose, patients were examined with methacholine BPT. After this procedure, the neuro-fuzzy system will make the final classification of asthma, COPD or normal lung function.

## Results

### ANN training results

Training algorithm for our ANN was Levenberg-Marquardt and it is trained and validated using estimation and validation data. The training data set contained 1000 tests, 800 of them were used for estimation, and other 200 for validation, which is presented in Table 2.

**Table 2:** Estimation and validation data for NN.

Number of reports	1000			
Estimation set size	800			

Validation set size	200			

**ESTIMATION SET**				

	No. of reports	True diagnosis	False diagnosis	% of true diagnosis

Asthma	350	348	2	99.43%

COPD	350	349	1	99.71%

Healthy	100	100	0	100%

			Average:	**99.71%**
**VALIDATION SET**				

	No. of reports	True diagnosis	False diagnosis	% of true diagnosis

Asthma	74	72	2	97.30%

COPD	75	74	1	98.67%

Healthy	51	51	0	100%

			Average:	**98.65%**

In the table 3 are presented the results of testing recurrent NN architecture with no visible improvement in classification, but with lower time and memory performance. These results are obtained using Matlab tool Profiler.

**Table 3:** Comparison between Feed Forward and Layer Recurrent ANN.

	Time to train with LMA [s]	Peak memory consumption [MB]
Feed Forward ANN	7.6	2.3

Layer Recurrent ANN	8.3	4.6

### System validation results

Our system is validated on 455 patients. Percentages of hits and misses of the neuro-fuzzy system in classification of asthmatics, COPD and healthy patients involved in this study are presented in table 4.

**Table 4:** Effectiveness of the neuro-fuzzy system in classification of asthma, COPD and healthy subjects.

Total population	Σ 455	Asthma	COPD	Healthy subjects	Average Accuracy
Asthma	Σ 170	169	0	1	99.41 %

COPD	Σ 248	1	246	1	99.19 %

Healthy subjects	Σ 37	0	0	37	100 %

**Figure 3:**
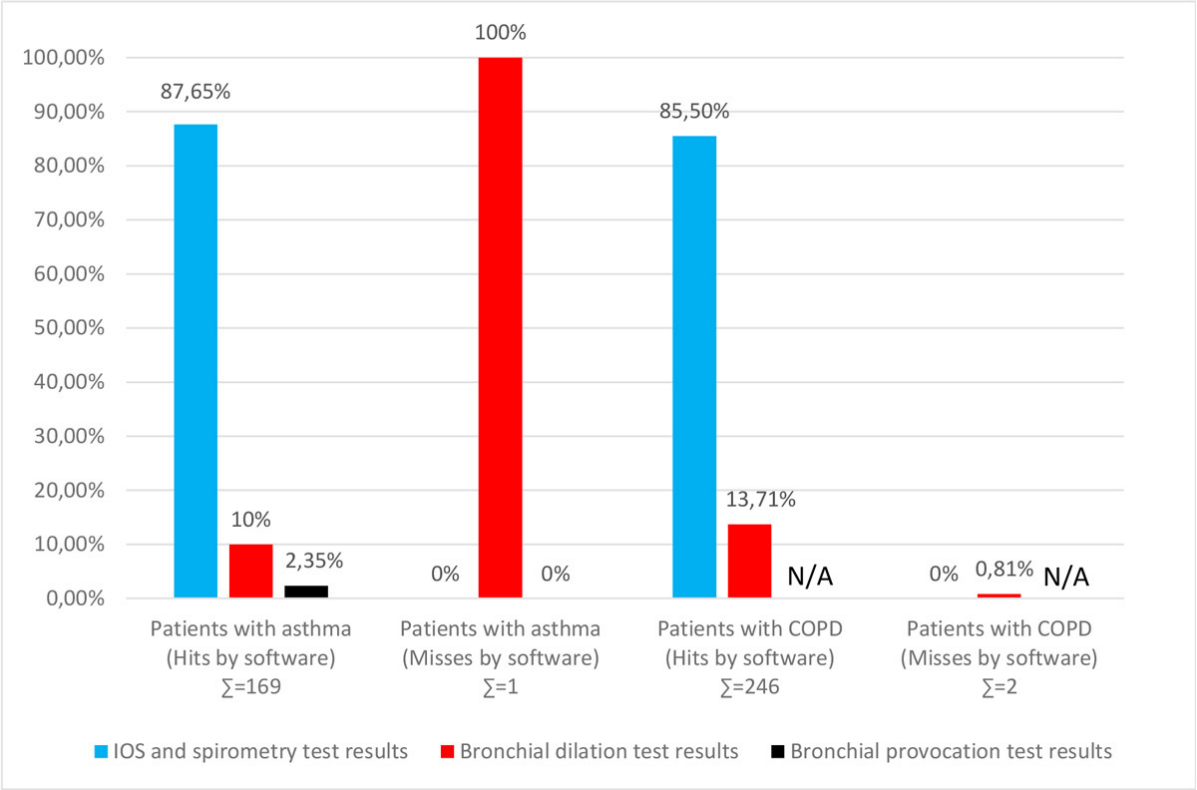
**Hits and misses of neuro-fuzzy system at each single step of classification in asthmatics and COPD patients**.

Results of neuro-fuzzy system at every step of classification in asthmatic and COPD patients are presented in Figure 3. Due to possibility of obtaining particular results for static and dynamic assessment of the patient, each step of neuro-fuzzy system classification is presented. This way of multiple steps created the possibility to compare our results with the results of other authors who have used only a static assessment of the patient during diagnosis of respiratory diseases.

As seen in Table 4 and Figure 3, the neuro-fuzzy system performed correct classification of disease for 452 participants, while for 3 participants performed incorrect classification of disease.

From total sum of 170 asthmatic patients the software correctly classified 149 patients after IOS and spirometry test results, 17 patients after BDT results, 4 patients after BPT results, while incorrectly classified 1 patient after BDT results.

From total sum of 248 COPD patients the software correctly classified 212 patients after IOS and spirometry test results, 34 patients after BDT results, while incorrectly classified 2 patients after BDT results.

It also may be noted that the software classified majority of the patients (over 85%) instantly upon the completion of standard tests (spirometry and IOS), without a single failure.

Full confusion matrix for performance of implemented algorithm is presented in table 5.

**Table 5:** Confusion matrix for performance of implemented algorithm.

	Total population 455	Condition positive	Condition negative	Prevalence 91.64%	
**Test outcome**	**Test outcome positive**	416	0	**Positive predictive value** 100%	**False discovery rate** 0.00%

	**Test outcome negative**	2	37	**False omission rate** 5.13%	**Negative predictive value** 94.87%

		**True positive rate** 99.52%	**False positive rate** 0.00%	**Accuracy** 99.56%	

		**False negative rate** 0.48%	**True negative rate** 100%		

The best and final results are achieved only after completing the entire process of classification of disease, i.e. after obtaining a complete dynamic assessment of the patient.

## Discussion

When making diagnosis of a respiratory disease, physicians use results obtained from spirometry and IOS. These results show that there is a correlation between parameters obtained by spirometry and IOS. In 2011, the Mehdi Nikkhah et al [35] compared methods of spirometry and IOS measurements in diagnosis of asthma and COPD patients. Furthermore, they demonstrated a correlation between IOS parameters and FEV1 parameter in asthmatics. They had shown that only R5 had a correlation with FEV1 in COPD patients, and that the progress of COPD disease is closely associated with the R5 parameter. Also, they had shown that in the case of their subjects sensitivity of X5 parameter in COPD patients is 76%, whereas in asthmatics sensitivity of R20 is 77%. In their studies, Kolsum et al in 2008 [36] and Song et al in 2009 [38] had already shown the correlation between R5 and X5 with FEV1. Since studies have shown that the combination of spirometry and IOS measurements achieve more successful diagnosis, the method which we developed for classification of asthma and COPD is using both type of measurements.

In 2009, Winkler et al [15] were able to diagnose 87-94% of asthma and COPD patients only when using different measurement methods on IOS. The same year Chakraborty et al [39] presented an intelligent diagnostic system for bronchial asthma based on symptoms and questions to which patients responded and obtained 90.03% accuracy in diagnosing asthma. In 2004, M. Barua et al [14] presented a system based on trained neural networks that uses the results of measurements performed by IOS for classification of asthma. They tested the system on 131 patients and obtained a 98% exact classification with known patterns, while their neural network, when working with unknown patterns, confirmed exact classification in 61% of cases. In our study, from a total sum of first time entered patients, after using only standard tests, the correct classification of patients was achieved in 87.65% of cases of asthma and 85.50% of COPD. If we use BDT and/or BPT suggested by neuro-fuzzy system, then we achieve correct classification in 99.41% of cases of asthma and 99.19% of COPD patients.

In 2006, Price D.B. et al [40] developed a tool in the form of a quiz based on the questions in order to recognize COPD in smokers and suggested further testing, while in the same year Tinkelman DG et al [41] further expanded their diagnostic tool for asthma and COPD. They tested it on 597 patients and obtained a sensitivity of 72.0% and specificity of 82.7%. While testing developed neuro-fuzzy system on our 455 study patients, we obtained sensitivity of 99.28% and specificity of 100%.

Tinkelman DG et al also presented the degrees in which the diagnosis of asthma and COPD are usually established, in the same way as presented in Figure 3 of our study.

In 2008, E. Meraz et al [17] and in 2009 N. Hafezi et al [18], based on known equivalent electrical models of lungs and their values specified for the healthy and diseased patients [42-49], developed a computational tool that classifies respiratory diseases in children by using IOS results. The advantage of our previous research [50-52] and new solution with respect to the one presented by E. Meraz et al [17] and N. Hafezi et al [18] is that our solution uses a combination of spirometry and IOS classification test results, which in the very beginning enables more accurate classification. Also, for the classification of diseases, in addition to the results obtained by using spirometry and IOS, symptoms according to GINA and GOLD rules as well as bronchiodilatory tests are necessary for proper classification of asthma and COPD. In this way, we get a complete patient's dynamic assessment, as opposed to the solution that provides a static assessment of the patient. The solution presented by Meraz [17] and Hafezi [18] is based on the equivalent electrical models of lungs, and those values are obtained from previous studies where age and race of the patients have to be taken into account, which is not the case in our solution.

In 2007, G. Coppin et al [53] introduced a computer system based on neural networks that detects emphysema using digital x-rays performed on patients with COPD, where they had accuracy of 90% on 161 subjects. This can be a great tool in combination with neuro-fuzzy system presented in our study.

In the future work, the aim of the researchers is to develop rules of fuzzy logic and neural network training for other respiratory diseases that can be determined on the basis of lung function tests.

## Conclusions

In this study we presented a neuro-fuzzy system for classification of asthma and chronic obstructive pulmonary disease (COPD). According to GINA and GOLD guidelines we defined fuzzy rules and neural network parameters. ANN of system was trained on more than one thousand medical reports obtained from database of the company CareFusion. Implemented neuro-fuzzy system was validated on 455 patients by physicians from the Clinical Centre University of Sarajevo. All patients were separated into two groups, healthy and diseased. Diseased subjects were separated into two subgroups, asthmatics and COPD patients. Out of 170 asthmatic patients, neuro-fuzzy system correctly classified 99.41% of patients. In addition, out of 248 COPD patients 99.19% were correctly classified. The system was 100% correct on 37 patients with normal lung function. Based on our neuro-fuzzy system we obtained sensitivity of 99.28% and specificity of 100% in asthma and COPD classification. These results have been achieved due to the fact that in our neuro-fuzzy system are also implemented all recommendations of GINA and GOLD necessary for classification of asthma and COPD. Also, as shown in the results, in the process of establishing the final diagnosis, complete dynamic assessment of the patient is obtained, as opposed to the solution that provides a static assessment of the patient.

## Competing interests

The authors declare that they have no competing interests.

## Authors' contributions

Almir Badnjevic obtained reports for training neural network, and together with Mario Cifrek developed fuzzy rules and neural network. Dinko Osmankovic helped in training of neural network and together with Almir and Mario they implemented neuro-fuzzy system. Dragan Koruga validated the system on Clinical centre University of Sarajevo. Almir Badnjevic and Mario Cifrek wrote an article.

## Declarations

Publication of this article was funded by authors.

This article has been published as part of *BMC Medical Informatics and Decision Making *Volume 15 Supplement 3, 2015: Multidimensional, multidiscipline and shared decision making in healthcare and eHealth. The full contents of the supplement are available online at http://www.biomedcentral.com/bmcmedinformdecismak/supplements/15/S3.
